# Insulin use and persistence in patients with type 2 diabetes adding mealtime insulin to a basal regimen: a retrospective database analysis

**DOI:** 10.1186/1472-6823-11-3

**Published:** 2011-01-12

**Authors:** Machaon M Bonafede, Anupama Kalsekar, Manjiri Pawaskar, Kimberly M Ruiz, Amelito M Torres, Karen R Kelly, Suellen M Curkendall

**Affiliations:** 1 Outcomes Research, Thomson Reuters Inc, 37 Lowell Street, Andover MA 01810, USA; 2 Current Address: Global Health Economics and Outcomes Research, Bristol Myers Squibb, PO Box 4000, Province Line Road, Princeton, NJ 08543, USA; 3 Global Health Outcomes: Endocrine/Obesity/Metabolism, Eli Lilly and Company, Lilly Technology Center, Indianapolis, IN 46285, USA; 4 Outcomes Research, Thomson Reuters Inc, 4301 Connecticut Ave NW, Suite 330. Washington DC 20008, USA; 5 Current Address: Healthcare Research and Analytics, GE Healthcare, 101 Cargenie Center, Princeton, NJ 08540, USA; 6 Diabetes and Devices Platform, Eli Lilly and Company, Lilly Technology Center, Indianapolis, IN 46202, USA

## Abstract

**Background:**

The objective of this study was to characterize insulin use and examine factors associated with persistence to mealtime insulin among patients with type 2 diabetes (T2D) on stable basal insulin therapy initiating mealtime insulin therapy.

**Methods:**

Insulin use among patients with T2D initiating mealtime insulin was investigated using Thomson Reuters MarketScan^® ^research databases from July 2001 through September 2006. The first mealtime insulin claim preceded by 6 months with 2 claims for basal insulin was used as the index event. A total of 21 months of continuous health plan enrollment was required. Patients were required to have a second mealtime insulin claim during the 12-month follow-up period. Persistence measure 1 defined non-persistence as the presence of a 90-day gap in mealtime insulin claims, effective the date of the last claim prior to the gap. Persistence measure 2 required 1 claim per quarter to be persistent. Risk factors for non-persistence were assessed using logistic regression.

**Results:**

Patients initiating mealtime insulin (n = 4752; 51% male, mean age = 60.3 years) primarily used vial/syringe (87%) and insulin analogs (60%). Patients filled a median of 2, 3, and 4 mealtime insulin claims at 3, 6, and 12 months, respectively, with a median time of 76 days between refills. According to measure 1, persistence to mealtime insulin was 40.7%, 30.2%, and 19.1% at 3, 6, and 12 months, respectively. Results for measure 2 were considerably higher: 74.3%, 55.3%, and 42.2% of patients were persistent at 3, 6, and 12 months, respectively. Initiating mealtime insulin with human insulin was a risk factor for non-persistence by both measures (OR < 0.80, p < 0.01). Additional predictors of non-persistence at 12 months included elderly age, increased insulin copayment, mental health comorbidity, and polypharmacy (p < 0.05 for all).

**Conclusions:**

Mealtime insulin use and persistence were both considerably lower than expected, and were significantly lower for human insulin compared to analogs.

## Background

For many patients with type 2 diabetes, initiation of insulin therapy marks the transition of their diabetes condition into a more severe disease state with the potential for more complications. The goal of type 2 diabetes treatment is to maintain a glycosylated hemoglobin (HbA1c) of < 7%, a goal that has been made easier through advances in insulin types and delivery options [1]. However, significant barriers still remain in physicians' and patients' minds regarding insulin use. The Diabetes Control and Complications Trial showed that patients treated with intensive insulin therapy demonstrated a 3-fold increased risk of a severe hypoglycemia event, a finding that physicians are aware of, according to Meece [2]. This awareness can therefore be a factor in clinical inertia for intensifying insulin therapy when patients are no longer controlled with basal insulin [3,4]. Patient fears regarding possible adverse health consequences associated with intensifying insulin therapy, such as weight gain or hypoglycemia, also factor into adherence to a treatment regimen including mealtime insulin. For most patients, the addition of mealtime insulin also requires insulin administration outside the home, which may have significant lifestyle implications due to public insulin use and inconveniences associated with carrying and storing insulin. Persistence with insulin is often low [5]; 16% to 49% of patients are persistent at 6 to 12 months [5-8]. Research suggests that medication regimen complexity [7] and higher frequency dosing schedules [9,10] are barriers to taking insulin. Thus, several treatment-related factors may be significant barriers to successful long-term treatment for some patients.

The primary objective of this study was to describe insulin use among patients adding mealtime insulin to their basal insulin regimen. The secondary objective of this study was to describe persistence to mealtime insulin and determine risk factors for poor persistence with insulin therapy. This study employed a retrospective approach using administrative claims data.

## Methods

Two Thomson Reuters MarketScan^® ^research databases were used in this study: the Commercial Claims and Encounters (Commercial) database and the Medicare Supplemental and Coordination of Benefits (Medicare Supplemental) Database. The Commercial database contains the inpatient, outpatient, emergency room, and outpatient prescription drug experience of several million individuals and their dependents in the United States. The overall database includes individuals from over 100 self-insured large employers and health plans. The Medicare Supplemental database contains the healthcare experience of individuals with Medicare supplemental insurance paid for by employers. The methods used in this current study were employed in a similar analysis of insulin naïve patients with type 2 diabetes using the same datasource and study design [11].

### Inclusion criteria

**Figure 1:**
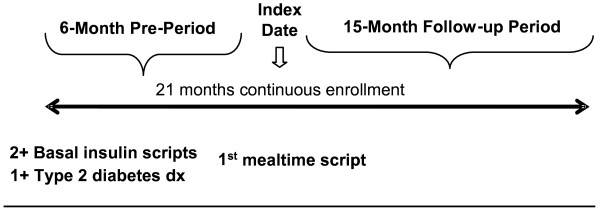
**Study Period**.

Patients were new mealtime insulin initiators between July 1, 2001 and December 31, 2006 (index date). A 6-month pre-period was used to establish new mealtime insulin use, a diagnosis of type 2 diabetes (identified by ICD-9-CM codes), and the presence of stable insulin therapy, as evidenced by having at least 2 claims for a basal insulin therapy in the 6 months preceding the index date. Patients had to be enrolled in a qualified health plan with concurrent continuous pharmacy enrollment for a 21-month period spanning the 6-month pre-index period through the 15-month study period between January 1, 2001 and March 31, 2008. Figure 1 illustrates the study period.

### Exclusion criteria

Patients were excluded if they had evidence of gestational diabetes (ICD-9-CM code 648.8×), type 1 diabetes (ICD-9-CM code 250. × 1 or 250. × 3, or DRG 295), or used inhaled insulin or an insulin pump anytime in the observation period. Patients were also excluded if they used an insulin mixture in the post-index period. Patients with only 1 mealtime insulin claim were also excluded from this analysis.

### Insulin Use Variables

Index insulin characteristics such as insulin type (human versus analog) and administration (pen versus vial/syringe) were reported. Examples of each insulin type are shown in table 1. Insulin use variables included:

**Table 1:** Insulin Type Classification

Class	Generic Name	Brand Name	Type
Basal	Insulin Human Isophane (NPH)	HUMULIN N NOVOLIN N RELION/NOVOLIN N INSULATARD	Human

Basal	Insulin Human Zinc (Lente)	HUMULIN L NOVOLIN L	Human

Basal	Insulin Human Zinc, Extended (Ultralente)	HUMULIN U	Human

Basal	Insulin Detemir	LEVEMIR	Analog

Basal	Insulin Glargine, Recombinant	LANTUS	Analog

Insulin Mixtures	Insulin Human Isophane (NPH)/Insulin Human Regular	HUMULIN 50/50 HUMULIN 70/30	Human

Insulin Mixtures	Insulin Human Isophane (NPH)/Insulin Human Regular	NOVOLIN 70/30 RELION NOVOLIN 70/30	Human

Insulin Mixtures	Insulin Lispro/Insulin Lispro Protamine	HUMALOG MIX 50/50 HUMALOG MIX 75/25	Analog

Insulin Mixtures	Insulin Aspart/Insulin Aspart Protamine	NOVOLOG MIX 70/30	Analog

Mealtime	Insulin Aspart, Recombinant	NOVOLOG	Analog

Mealtime	Insulin Glulisine	APIDRA	Analog

Mealtime	Insulin Lispro, Recombinant	HUMALOG LISPRO-PFC	Analog

Mealtime	Insulin Human Regular	HUMULIN R NOVOLIN R RELION/NOVOLIN R	Human

Mealtime	Insulin Human Regular, Buffered	HUMULIN BR VELOSULIN BR	Human

1) Number of prescription claims for mealtime insulin at 3, 6, and 12 months

2) Number of prescription claims for basal insulin at 3, 6, and 12 months

3) Average daily insulin supply calculated at 3, 6, and 12 months post-index date by calculating the total insulin supply in ml dispensed and dividing by the number of days in that period.

4) Average time between refills of index insulin

5) Proportion that switched to a different insulin type or administration

6) Presence of a 90-day gap between insulin claims.

### Insulin Persistence Measures

This study evaluated persistence with mealtime insulin at 12 months, defined as the time from mealtime insulin initiation to discontinuation. Persistence was evaluated at 12 months by the absence of gaps between refills (measure 1) or the number of refills within a pre-specified period (measure 2). Measure 2 was designed as a more lenient sensitivity analysis to measure 1, which is a standard definition of persistence [12]. Under measure 1, patients were considered persistent at 3 months if they did not have a 90-day gap in index insulin claims that started prior to the end of the third month. Patients were considered persistent at 6 and 12 months if they did not have 90-day gaps prior to the end of the relevant time periods. For example, if a patient had an index claim on January 1 and had refills on March 15, May 1, and July 15 and no others refills, then the patient would be persistent at 6 months (due to no 90-day gaps prior to July 1) but not at 12 months.

Measure 2 was defined as follows:

• Persistent at 3 months

◦ At least 2 mealtime insulin claims in first 4 months

• Persistent at 6 months

◦ At least 3 mealtime insulin claims in first 7 months

▪ At least one mealtime insulin claim in months 1-3, and

▪ At least one mealtime insulin claim in months 4-6

• Persistent at 12 months

◦ At least 4 mealtime insulin claims in first 12 months

▪ At least one mealtime insulin claim in months 1-3, and

▪ At least one mealtime insulin claim in months 4-6, and

▪ At least one mealtime insulin claim in months 7-9, and

▪ At least one mealtime insulin claim in months 10-12

For measure 2, counts of mealtime insulin claims include the index claim. For example, for a patient to be persistent at 3 months, they would need to fill at least 1 additional mealtime insulin claim within 4 months following their index claim. Non-persistence, as determined by these measures, does not imply a permanent discontinuation from insulin and may be a marker for intermittent use.

Persistent and non-persistent patients were compared using standard tests of statistical significance. Chi-square tests were used to evaluate the statistical significance of differences for categorical variables; two-tailed t-tests and analysis of variance were used for continuous variables. A series of logistic regression models were used to examine factors associated with being persistent with insulin therapy at 12 months. Factors included age, gender, location, type of insurance, insulin type, insulin administration mode, Deyo Charlson Comorbidity Index [13], presence of hospitalization, presence of emergency room visit, presence of diabetes complications, presence of macrovascular complications, presence of mental health disorders, count of other diabetes medications and average copayment per claim of insulin. An alpha of 0.05 was used for all analyses.

## Results

A total of 294,769 patients who had 1 or more mealtime insulin claim were identified during the study period. Of this total, 66,805 (22%) patients had a type 2 diabetes diagnosis and continuous enrollment. Requiring 2 claims of basal insulin in the pre-period brought the sample to 19,668. Evidence of type I diabetes caused 13,184 patients to be excluded. An additional 27 patients were excluded for a diagnosis of gestational diabetes or for using inhaled insulin or an insulin pump. This left a total of 6,457 patients on stable basal insulin who initiated a mealtime insulin regimen. Seven hundred forty-eight (approximately 12%) of those patients were excluded from further analysis because they had a claim for an insulin mixture in the post-index period. Of the remaining 5,709 patients, 957 (14.8%) had a single mealtime claim, leaving 4,752 as the final sample size of patients with 2 or more mealtime insulin claims in the post-index period.

Table 2 contains the insulin use characteristics of the study sample. The average time between refills over 12 months was 94.5 days (median: 75.5 days). The majority of the sample (80.9%) had a 90-day gap in the 1-year following initiation of mealtime insulin. The mean time to the start of the 90-day gap was 67.6 days, and the time between the start of the 90-day gap and the next claim was 160.0 days. The majority of patients (86.6%) with a 90-day gap had a claim following the gap. Persistence was 40.7% at 3 months, 30.2% at 6 months, and 19.1% at 12 months using measure 1. Persistence was 74.3% at 3 months, 55.3% at 6 months, and 42.2% at 12 months using measure 2.

**Table 2:** Insulin Use Characteristics

	Mean	Median	Interquartile Range
**Number of claims: Mealtime**			

3 months	1.95	2	(1, 2)

6 months	3.11	3	(2, 4)

12 months	5.32	4	(3, 7)

**Number of claims: Basal**			

3 months	1.93	2	(1, 3)

6 months	3.65	3	(2, 5)

12 months	6.95	6	(4, 10)

**Number of claims: Total**			

3 months	3.89	4	(3, 5)

6 months	6.76	6	(4, 9)

12 months	12.27	11	(8, 15)

**Quantity per day (mL): Mealtime**			

3 months	0.23	0.22	(0.11, 0.33)

6 months	0.19	0.17	(0.11, 0.22)

12 months	0.16	0.14	(0.08, 0.21)

**Quantity per day (mL): Basal**			

3 months	0.22	0.22	(0.11, 0.33)

6 months	0.21	0.17	(0.11, 0.28)

12 months	0.19	0.16	(0.11, 0.27)

**Quantity per day (mL): Total**			

3 months	0.45	0.44	(0.33, 0.56)

6 months	0.39	0.33	(0.28, 0.50)

12 months	0.35	0.33	(0.22, 0.44)

**Time between mealtime refills**			

12 months	94.5	75.5	(48, 116)

**Time from index to start of 90-day gap among patients with a 90-day gap**	*N = 3843 (80.9% of total sample)*		

12 months	67.6	25	(1, 95)

**Time between start of 90-day gap and next mealtime script among patients with a mealtime script following a 90-day gap**	*N = 3335 (86.8% of patients with a 90-day gap)*		

12 months	160.0	135	(107, 192)

In terms of demographics, there were several statistically significant differences between persistent and non-persistent patients; however, several of these differences were not of practical importance. For example, persistent patients were statistically but not substantively younger than non-persistent patients (measure 1: 58.6 versus 60.7, p < 0.0001; measure 2: 59.7 versus 60.8 years, p = 0.0043). There were also numerous statistically significant differences between persistent and non-persistent patients in terms of clinical characteristics. Table 3 contains comparisons of demographic and clinical characteristics stratified by persistence status. The mean Deyo Charlson Comorbidity Index score was lower among persistent patients than non-persistent patients (measure 1: 2.05 versus 2.22, p = 0.015; measure 2: 2.10 versus 2.25, p = 0.004). Persistent patients had lower counts of concomitant diabetes medication classes than non-persistent patients (measure 1: 0.86 versus 1.05 classes, p < 0.0001; measure 2: 0.94 versus 1.07 classes, p < 0.0001). Persistent patients were also less likely than non-persistent patients to have diabetes complications (measure 1: 28.5% versus 32.6%, p = 0.017; measure 2: 29.9% versus 33.2%, p = 0.014), macrovascular complications (measure 1: 48.4% versus 50.7%, p = 0.215; measure 2: 48.5% versus 51.6%, p = 0.037), and mental health disorders (measure 1: 5.6% versus 7.4%, p = 0.056; measure 2: 6.2% versus 7.7%, p = 0.040).

**Table 3:** Demographic and Clinical Characteristics

	Persistence Measure 1			Persistence Measure 2			Total
	**Persistent**	**Non-persistent**		**Persistent**	**Non-persistent**		

	**N = 909**	**N = 3,843**	**p-value**	**N = 2,007**	**N = 2,745**	**p-value**	**N = 4,752**

**Gender**: Female (%)	47.85	49.15	0.481	47.98	49.58	0.276	48.91

**Age (mean [SD])**	58.6 (11.8)	60.7 (12.3)	<0.0001	59.7 (12.1)	60.8 (12.3)	0.0043	60.3 (12.2)

**Age Group (%)**							

<18	0.22	0.16	<0.0001	0.20	0.15	0.069	0.17

18-34	1.76	1.43		1.54	1.46		1.49

35-44	7.92	6.48		6.78	6.74		6.76

45-54	26.07	22.85		24.76	22.51		23.46

55-64	36.74	33.49		35.33	33.22		34.11

65-74	16.94	20.66		18.73	20.84		19.95

75 and older	10.34	14.94		12.66	15.08		14.06

**Location (%)**							

Urban	77.34	80.61	0.018	79.02	80.69	0.091	79.99

Rural	22.44	18.79		20.63	18.65		19.49

Unknown	0.22	0.60		0.35	0.66		0.53

**Insulin Type- index claim (%)**							
Human	37.62	40.62	0.097	36.92	42.33	<0.0001	40.05

Analog	62.38	59.38		63.08	57.67		59.95

**Insulin Administration- index claim (%)**							

Vials and Syringes	88.01	86.76	0.312	86.70	87.21	0.601	86.99

Pens	11.99	13.24		13.30	12.79		13.01

**Change in Index Insulin**							

Human vs. Analog Change (%)	26.84	28.57	0.298	26.46	29.54	0.020	28.24

Mean time to change (SD)	52.79 (86.30)	57.50 (81.95)	0.421	56.14 (83.32)	56.98 (82.42)	0.856	56.65 (82.75)

Pen vs. vial/syringe change (%)	4.40	3.54	0.216	4.68	2.99	0.002	3.70

Mean time to change (SD)	145.1 (107.34)	154.13 (109.70)	0.646	150.64 (113.69)	153.73 (103.88)	0.852	52.08 (108.93)

**Insulin Use: 12 months (mean [SD])**							

Mealtime Insulin Claims	9.99 (3.36)	4.21 (2.06)	<0.0001	7.90 (3.30)	3.43 (1.50)	<0.0001	5.32 (3.28)

Basal Insulin Claims	9.64 (4.41)	6.32 (3.65)	<0.0001	8.17 (4.24)	6.06 (3.60)	<0.0001	6.95 (4.02)

Total Insulin Claims	19.63 (6.42)	10.53 (4.60)	<0.0001	16.07 (6.42)	9.49 (4.13)	<0.0001	12.27 (6.15)

Mealtime Insulin quantity per day (ml)	0.30 (0.11)	0.13 (0.06)	<0.0001	0.23 (0.10)	0.10 (0.05)	<0.0001	0.16 (0.10)

Basal Insulin quantity per day (ml)	0.27 (0.13)	0.18 (0.10)	<0.0001	0.23 (0.12)	0.17 (0.10)	<0.0001	0.19 (0.11)

Total Insulin quantity per day (ml)	0.56 (0.19)	0.30 (0.13)	<0.0001	0.46 (0.19)	0.27 (0.12)	<0.0001	0.35 (0.18)

Days between Mealtime Insulin Claims	42.44 (13.68)	106.77 (69.27)	<0.0001	58.06 (23.98)	121.09 (76.10)	<0.0001	94.47 (67.50)

**Concomitant Medications Count**							

Diabetes medications	0.87 (0.91)	1.07 (0.99)	<0.0001	0.95 (0.93)	1.09 (1.01)	<0.0001	1.03 (0.98)

Cardiovascular medications	4.07 (2.36)	4.23 (2.49)	0.072	4.25 (2.42)	4.17 (2.49)	0.265	4.20 (2.46)

**Copayment Burden**							

Total cost of pharmacy claims	$6,122 (5657)	$5,663 (4567)	0.009	$6,221 (5310)	$5,408 (4355)	<0.0001	$5,751 (4798)

Copay per insulin claim	$15.68 (12.21)	$18.41 (29.82)	0.007	$16.63 (13.20)	$18.81 (34.17)	0.007	$17.89 (27.37)

**Deyo Charlson Comorbidity Index (mean)**	2.05 (1.76)	2.22 (1.82)	0.015	2.10 (1.73)	2.25 (1.86)	0.004	2.19 (1.81)

**Comorbidities (%)**							

Diabetes complications	28.49	32.58	0.017	29.85	33.22	0.014	31.80

Macrovascular complications	48.40	50.69	0.215	48.48	51.55	0.037	50.25

Any mental health	5.61	7.42	0.056	6.18	7.72	0.040	7.70

**Diabetes-Related Events**							

Inpatient Admissions (%)	0.55	1.09	0.294	0.70	1.20	0.124	0.99

Mean length of stay	4.2 (1.48)	7.10 (6.59)	0.337	7.64 (4.81)	6.42 (6.87)	0.550	6.79 (6.30)

ER Use (%)	0.66	0.62	0.903	0.75	0.55	0.388	0.63

**Cardiovascular-Related Events**							

Inpatient Admissions (%)	4.18	5.54	0.381	5.28	5.28	0.675	5.28

Mean length of stay	2.87 (3.83)	4.56 (6.53)	0.124	3.57 (3.15)	4.83 (7.70)	0.112	4.30 (6.22)

ER Use (%)	2.09	2.2%	0.785	2.14	2.26	0.788	2.21

Abbreviation: SD = standard deviation; ER = Emergency Room

Persistent patients were less likely than non-persistent patients to use human insulin at index (36.9% versus 42.3%, p < 0.0001) as defined by measure 2. As expected (by definition of persistence), the number of claims and quantity per day was higher among persistent patients, and time between insulin claims was lower among persistent patients, compared to non-persistent patients. The mean total cost of claims was higher among persistent patients than non-persistent patients (measure 1: $6,122.81 versus $5,663.12, p = 0.009; measure 2: $6,220.88 versus $5,407.54, p < 0.0001) while the average copayment per insulin claim was lower (measure 1: $15.68 versus $18.41, p = 0.007; measure 2: $16.63 versus $18.81, p = 0.007).

### Multivariate Results: Persistence at 12 months

Multivariate results are summarized in Table 4. Gender was not statistically significantly associated with persistence. Compared to patients 45 to 54 years, those over age 65 were statistically significantly less likely to be persistent at 12 months. Living in a rural location was associated with increased persistence by measure 1 (OR = 1.26 [95% CI: 1.05, 1.52], p = 0.013) but not by measure 2 (OR = 1.12 [95% CI: 0.97, 1.31], p = 0.129). A capitated insurance plan was also not statistically significantly associated with insulin persistence at 12 months.

**Table 4:** Multivariate Analysis

Logistic Regression				
**Sample Size (N = 4,752)**	**Persistence at 12 months: Measure 1**		**Persistence at 12 months: Measure 2**	

	**OR (95% CI)**	**p-value**	**OR (95% CI)**	**p-value**

Female	0.96 (0.83, 1.12)	0.62	0.94 (0.84, 1.06)	0.33

Age < 35	1.04 (0.60, 1.81)	0.89	1.02 (0.64, 1.62)	0.94

Age 35-44	1.06 (0.78, 1.43)	0.72	0.94 (0.73, 1.21)	0.62

Age 55-64	0.96 (0.79, 1.17)	0.69	0.97 (0.83, 1.14)	0.70

Age 65-74	0.73 (0.57, 0.92)	0.01	0.82 (0.68, 0.99)	0.03

Age >74	0.63 (0.48, 0.83)	0.00	0.81 (0.65, 0.99)	0.04

Region: North Central	0.84 (0.64, 1.10)	0.20	0.85 (0.68, 1.06)	0.15

Region: South	0.85 (0.65, 1.11)	0.24	0.81 (0.65, 1.01)	0.06

Region: West	0.80 (0.60, 1.07)	0.13	0.81 (0.64, 1.02)	0.07

Region: Unknown	0.31 (0.07, 1.33)	0.12	0.42 (0.17, 1.02)	0.06

Rural	1.26 (1.05, 1.52)	0.01	1.12 (0.97, 1.31)	0.13

Capitated Insurance	1.18 (0.98, 1.43)	0.08	1.04 (0.89, 1.21)	0.62

Index: Human	0.80 (0.68, 0.95)	0.01	0.77 (0.67, 0.87)	<0.0001

Index: Pen	0.86 (0.68, 1.08)	0.21	0.98 (0.82, 1.17)	0.79

Deyo Charlson Com. Index	0.99 (0.94, 1.04)	0.67	0.97 (0.93, 1.01)	0.11

ER Admissions	1.13 (0.71, 1.81)	0.60	1.18 (0.82, 1.70)	0.37

Inpatient Admissions	0.90 (0.62, 1.31)	0.58	0.95 (0.72, 1.26)	0.73

Diabetes Complications	0.89 (0.74, 1.07)	0.22	0.90 (0.78, 1.04)	0.16

Count of OAD Classes	0.99 (0.96, 1.03)	0.60	1.04 (1.01, 1.06)	0.01

Macrovascular Complications	1.03 (0.87, 1.21)	0.75	0.89 (0.78, 1.01)	0.08

Mental Health Disorders	0.76 (0.55, 1.04)	0.08	0.81 (0.64, 1.02)	0.08

Average Copay for Insulin	0.99 (0.98, 0.99)	<0.0001	0.99 (0.99, 0.99)	<0.0001

Reference Groups: Age 45-54, Region: NorthEast, Non-Capitated Insurance				

Abbreviation: Com Inex = Comorbidity Index; OAD = oral antidiabetic agents; OR = odds ratio;

CI = Confidence Interval

The use of human insulin was associated with decreased odds by persistence measures 1 and 2 (OR = 0.80 [95% CI: 0.68, 0.95] and 0.77 [95% CI: 0.67, 0.87], p < 0.01 for both). The use of an insulin pen was not statistically significantly associated with persistence at 12 months. None of the clinical characteristics were associated with insulin persistence at 12 months. The count of oral antidiabetic (OAD) agents was statistically significant by measure 2 (OR = 1.04 [95% CI: 1.00, 1.06], p = 0.01), but not by measure 1 (OR = 0.99 [95% CI: 0.96, 1.03], p = 0.601). The average copayment for insulin was a predictor of non-persistence by persistence measures 1 and 2 (OR = 0.99, p < 0.0001 for both).

## Discussion

The majority of patients initiating mealtime insulin had a 90-day gap in mealtime insulin claims (81%) during the year following their first mealtime insulin claim. According to Sikka and colleagues, patients filling claims following an allowable gap are still considered non-persistent [14]. However, the presence of a 90-day gap is not a measure of complete discontinuation; 87% of patients with a 90-day gap had a mealtime insulin claim following the gap. Of the 3,843 patients with a 90-day claim gap, nearly half (46.2%) had a gap immediately following their index claim. Using a 90-day gap to define insulin persistence increases the likelihood of falsely classifying persistent patients as non-persistent. According to measure 2, which is more lenient and allows for sizeable gaps between the required number of insulin claims, less than half of patients (42%) were persistent at 12 months.

A significant number of patients who met the study inclusion and exclusion criteria (17%) were excluded because they only filled their index mealtime insulin claim. A single mealtime insulin claim may indicate a response to an acute hyperglycemic event and not represent a true intensification of diabetes management. Further investigation of these patients and their reasons for early discontinuation is warranted.

Several factors could be related to the findings regarding insulin type (analog versus human) and delivery (pen versus vial/syringe). Previous research has found that a dislike of injections is related to discontinuing insulin use [8], which might imply that insulin pens may have better persistence than vial/syringe due to the somewhat different nature of the injection. However, this study failed to find a statistically significant relationship between insulin pen use and persistence, similar to the findings of Pawaskar and colleague [15]. This finding may be partly due to the availability of products and the study timeframe. At the same time, the greater dosing flexibility may have contributed to the consistent association between analog insulin and increased persistence. This current analysis could not, however, control for provider-related characteristics or preferences that may be related to insulin persistence and the use of human versus analog or pen versus vial/syringe insulin.

The finding that higher counts of concomitant diabetes medications were positively associated with insulin persistence is inconsistent with existing literature [10]. This association could be due, in part, to the fact that the use of additional classes of OAD agents are a result of healthcare utilization and monitoring. It is also possible that collinearity between the count of OAD classes and diabetes complications is interfering with the multivariate models. This may explain the change in direction from the univariate to the multivariate results.

Caution should be used when interpreting the results of persistency to insulin therapy. There is no validated measure available to measure insulin adherence and persistence. The presence of a claim gap (measure 1) is a commonly used measure for persistency [16]. The original formulation of measure 2 incorporated the current number of claims structure and a requirement of 0.10 mL index insulin per day, on average. Descriptive analyses of the average quantity of insulin per day did not provide a clear, clinically relevant cut-off that did not appear arbitrary. Descriptive analysis also indicated that the number of claims requirement, as opposed to the insulin quantity per day, largely determined persistence status. Both of these reasons support excluding insulin quantity per day from persistence determination.

By either measure, non-persistence indicates a significant break in a patient's use of mealtime insulin that is reflected both in time off of therapy and in average daily dose of insulin. Non-persistent patients had an average total daily insulin dose of 0.30 mL (non-persistent by measure 1) and 0.27 mL (non-persistent by measure 2), which included average daily mealtime insulin dose per day of 0.13 mL and 0.10 mL, respectively. While the recommended quantity of mealtime insulin per day differs by patient, these levels are significantly lower than would be expected in a group of patients with adequately managed type 2 diabetes using mealtime insulin [17,18].

The Thomson Reuters MarketScan^® ^research databases are comprised of only adjudicated claims; this minimizes, but not completely removes, the risk of incomplete, missing, or miscoded claims impacting the study findings. Errors present are likely random and independent of the study outcomes and cohorts, further minimizing their potential impact on the study. A related and significant limitation is the lack of records involving services that do not drive a service payment. Lab values (including hemoglobin A1C), patient biometric measures, and a record of free insulin samples are some of the key missing data fields. Further, the database contains very little information regarding physician behavior, training, or patient instructions, making it impossible to investigate or control for physician-level factors, including physician tendencies to prescribe more or less insulin per visit.

## Conclusions

In a cohort of patients intensifying their basal insulin therapy with the addition of mealtime insulin, use was lower than expected in terms of quantity per day, number of claims, and persistence. In Addition, persistence to mealtime insulin decreases over time. Persistence to human insulin was considerably lower than the analog insulin. Elderly age, human insulin use, increased average insulin copayment, mental health comorbidity, and polypharmacy were all consistent predictors of poorer persistence with mealtime insulin.

## Competing interests

The authors have no competing interests to report. This work was conducted by Thomson Reuters and funded by Eli Lilly. MB, KR and SC are employees of Thomson Reuters. AT was an employee of Thomson Reuters at the time the work was conducted. MP and KK are employees of Eli Lilly. AK was an employee of Eli Lilly at the time the work was conducted.

## Authors' contributions

All of the authors contributed to the study design, data interpretation, and discussion of study results. All authors approve of this manuscript and were active participants throughout the life of the study. MB was the principal investigator for the study. AK and MP developed the study concept and research questions. KK provided insight on the clinical relevance and appropriateness of the research questions and results. LT and KR constructed the analytic file used for the study, operationalizing study concepts and linking them to specific research questions. SC provided senior oversight and quality assurance. All of the authors read and approve of the final manuscript.

## Pre-publication history

The pre-publication history for this paper can be accessed here:

http://www.biomedcentral.com/1472-6823/11/3/prepub

## References

[B1] American Diabetes Association Standards of medical care in diabetes--2008 Diabetes Care 2008 31 suppl. 1 S12 S54 18165335 10.2337/dc08-S012

[B2] Meece J Dispelling myths and removing barriers about insulin in type 2 diabetes Diabetes Educ 2006 32 1 Suppl 9S 18S 10.1177/0145721705285638 16439485

[B3] Korytkowski M When oral agents fail: Practical barriers to starting insulin Int J Obes Relat Metab Disord 2002 26 S18 24 10.1038/sj.ijo.0802173 12174319

[B4] Riddle MC The underuse of insulin therapy in North America Diabetes Metab Res Rev 2002 18 3 Suppl S42 S49 10.1002/dmrr.277 12324985

[B5] Cramer JA A systematic review of adherence with medications for diabetes Diabetes Care 2004 27 1218 1224 10.2337/diacare.27.5.1218 15111553

[B6] Catalan VS Couture JA Lelorier J Predictors of persistence of use with the novel antidiabetic agent acarbose Arch Int Med 2001 161 1106 1112 10.1001/archinte.161.8.1106 11322845

[B7] Dailey G Kim MS Lian JF Patient compliance and persistence with antihyperglycemic drug regimens: evaluation of a Medicaid patients population with type 2 diabetes mellitus Clin Ther 2001 23 1311 1320 10.1016/S0149-2918(01)80110-7 11558867

[B8] Dezii CM Kawabata H Tran M Effects of once-daily and twice-daily dosing on adherence with prescribed glizipide oral therapy for type 2 diabetes South Med J 2002 95 68 71 11827247

[B9] Paes AH Bakker A Soe-Agnie CJ Impact of dosage frequency on patient compliance Diabetes Care 1997 20 10 15121517 10.2337/diacare.20.10.1512 9314626

[B10] Odegard PS Capoccia K Medication taking and diabetes: A systematic review of the literature Diabetes Educ 2007 33 1014 1029 10.1177/0145721707308407 18057270

[B11] Bonafede M Kalsekar A Pawaskar M Ruiz KM Torres AM Kelly KR Curkendall SM A retrospective database analysis of insulin use patterns in insulin-naïve patients with type 2 diabetes initiating basal insulin or mixtures Pat Pref Adh 2010 4 147 156 10.2147/ppa.s10467 PMC2898116 20622915

[B12] Peterson AM Nau DP Cramer JA Benner J Gwadry-Sridhar F Nichol M A Checklist for Medication Compliance and Persistence Studies Using Retrospective Databases Value in Health 2007 10 1 3 12 10.1111/j.1524-4733.2006.00139.x 17261111

[B13] Deyo RA Cherkin DC Ciol MA Adapting a clinical comorbidity index for use with ICD-9-CM administrative databases J Clin Epidemiol 1992 45 613 19 10.1016/0895-4356(92)90133-8 1607900

[B14] Sikka R Xia F Aubert RE Estimating Medication Persistency Using Administrative Claims Data Am J Manag Care 2005 11 449 457 16044982

[B15] Pawaskar MD Camacho FT Anderson RT Cobden D Joshi AV Balkrishnan R Health care costs and medication adherence associated with initiation of insulin pen therapy in medicaid-enrolled patients with type 2 diabetes: a retrospective database analysis Clin Ther 2007 29 1294 305 10.1016/j.clinthera.2007.07.007 18046929

[B16] Fabunmi R Nielsen LL Quimbo R Schroeder B Misurski D Wintle M Wade R Patient characteristics, drug adherence patterns, and hypoglycemia costs for patients with type 2 diabetes mellitus newly initiated on exenatide or insulin glargine Curr Med Res Opin 2009 25 3 777 786 10.1185/03007990802715199 19203299

[B17] Mayfield JA White RD Insulin therapy for type 2 diabetes: Rescue, augmentation, and replacement of beta-cell function Amer Fam Phys 2004 70 3 489 500 15317436

[B18] Yki-Järvinen H Combination therapies with insulin in type 2 diabetes Diabetes Care 2001 24 758 67 11315844 10.2337/diacare.24.4.758

